# Neoadjuvant cemiplimab to facilitate Mohs micrographic surgery tumor extirpation and reduce surgical morbidity

**DOI:** 10.1016/j.jdin.2025.09.014

**Published:** 2025-10-15

**Authors:** Marianne Cortes, Jorge O. Santana, John Strasswimmer

**Affiliations:** a Corewell Health Dermatology Program, Trenton, Michigan; b Johns Hopkins University, Baltimore, Maryland; c Florida Atlantic University- Charles E. Schmidt College of Medicine, Boca Raton, Florida; d Strasswimmer Mohs Surgery, Delray Beach, Florida

**Keywords:** SCC, advanced cutaneous squamous cell carcinoma, cutaneous oncology, cemiplimab, LIBTAYO, immunotherapy, anti-PD-1

## Clinical challenge and therapeutic pearl

Advanced cutaneous squamous cell carcinoma (aCSCC) is treated with immune checkpoint inhibitors (cemiplimab or pembrolizumab) that require 2 years of administration and have a significant risk of life-threatening autoimmune complications.^1^ Recently, neoadjuvant short-course therapy was used in a pilot study for aCSCC, but the patients still required general anesthesia in an operating room. In addition, 51% of the patients also received ongoing adjuvant immunotherapy or adjuvant postoperative radiation therapy.^2^ It is not clear whether short-course neoadjuvant immunotherapy alone might be sufficient to decrease the surgical burden while maintaining oncologic control. We present here the successful use of short-term immunotherapy to convert a nonresectable tumor into one that was able to be treated in-office under local anesthesia with a good long-term cure.

**Fig 1:**
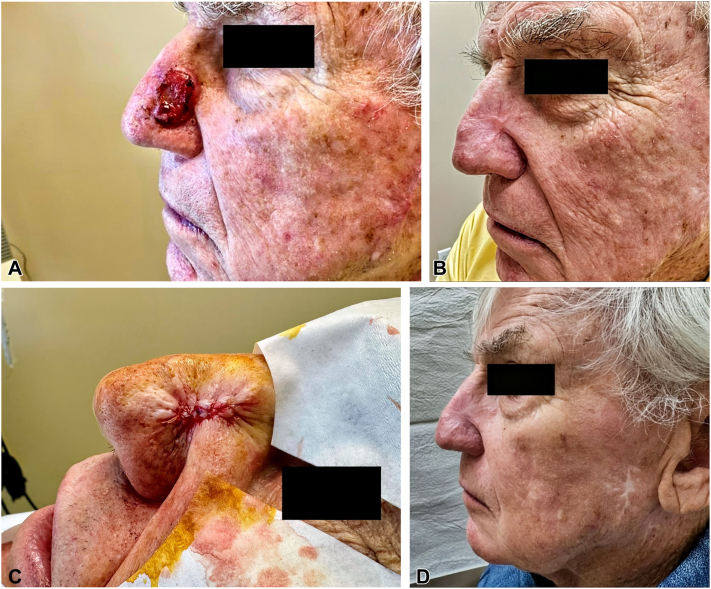
**A,** Advanced cutaneous squamous cell carcinoma at presentation. **B,** Following 6 cycles of neoadjuvant cemiplimab therapy. **C,** Mohs micrographic surgery and reconstruction. **D,** The 1-year follow-up after initial presentation.

An 88-year-old man with early dementia presented with a large aCSCC (American Joint Committee on Cancer stage III, Brigham and Women's Hospital staging system stage IIb) on the nasal sidewall (Fig 1, *A*). He was not a candidate for general anesthesia or for extended radiation sessions. We assessed that resection of the tumor will likely result in an extensive full-thickness defect, which could not be safely or ethically repaired under local anesthesia in the office. For this reason, we sought to determine whether a short course of immunotherapy might render this small enough for in-office reception and long-term management.

The patient was initiated on cemiplimab for 6 cycles. The tumor size decreased to the point where in-office resection under local anesthesia was possible (Fig 1, *B*). Tumor extirpation was achieved with stage I of Mohs micrographic surgery and reconstructed with a linear closure (Fig 1, *C*). In total, 22 months after presentation, there is no recurrence (Fig 1, *D*). This therapeutic approach may benefit other patients with challenging aCSCC.

## Conflicts of interest

Dr Strasswimmer works with the following companies as a consultant and as a clinical trials researcher: Regeneron Pharmaceuticals, Phio Pharmaceuticals, Alpha Tau Inc, Biofrontera Inc, Sun Pharmaceutical Inc. Other authors have nothing to disclose.
